# Lime pretreatment of sugar beet pulp and evaluation of synergy between ArfA, ManA and XynA from *Clostridium cellulovorans* on the pretreated substrate

**DOI:** 10.1007/s13205-011-0019-3

**Published:** 2011-08-19

**Authors:** Roselyn Dredge, Sarah E. Radloff, J. Susan van Dyk, Brett I. Pletschke

**Affiliations:** 1 Department of Biochemistry, Microbiology and Biotechnology, Rhodes University, Grahamstown, 6140 South Africa; 2 Department of Statistics, Rhodes University, Grahamstown, 6140 South Africa

**Keywords:** Arabinofuranosidase, Lignocellulose, Mannanase, Sugar beet pulp, Synergy, Xylanase

## Abstract

Sugar beet pulp (SBP) is a waste product from the sugar beet industry and could be used as a potential biomass feedstock for second generation biofuel technology. Pretreatment of SBP with ‘slake lime’ (calcium hydroxide) was investigated using a 2^3^ factorial design and the factors examined included lime loading, temperature and time. The pretreatment was evaluated for its ability to enhance enzymatic degradation using a combination of three hemicellulases, namely ArfA (an arabinofuranosidase), ManA (an *endo*-mannanase) and XynA (an *endo*-xylanase) from *C. cellulovorans* to determine the conditions under which optimal activity was facilitated. Optimal pretreatment conditions were found to be 0.4 g lime/g SBP, with 36 h digestion at 40 °C. The synergistic interactions between ArfA, ManA and XynA from *C. cellulovorans* were subsequently investigated on the pretreated SBP. The highest degree of synergy was observed at a protein ratio of 75% ArfA to 25% ManA, with a specific activity of 2.9 U/g protein. However, the highest activity was observed at 4.2 U/g protein at 100% ArfA. This study demonstrated that lime treatment enhanced enzymatic hydrolysis of SBP. The ArfA was the most effective hemicellulase for release of sugars from pretreated SBP, but the synergy with the ManA indicated that low levels of mannan in SBP were probably masking the access of the ArfA to its substrate. XynA displayed no synergy with the other two hemicellulases, indicating that the xylan in the SBP was not hampering the access of ArfA or ManA to their substrates and was not closely associated with the mannan and arabinan in the SBP.

## Introduction

Sugar beet (*Beta vulgaris*) is grown commercially for its high sucrose content and contributes to 35% of sucrose production worldwide (Foster et al. 2001). The residue remaining after the extraction of sucrose is known as sugar beet pulp (SBP). Current uses of SBP include use as an animal feed (Deaville et al. 1994; Serena and Knudsen 2007), as a pectin source for the food industry (Turquois et al. 1999) and in the production of paper (Vaccari et al. 2005). However, SBP could also be a potential source of renewable biomass for biofuel production (Foster et al. 2001).

The chemical composition of SBP, as reported in literature, reveals a large hemicellulose fraction (45–61% dry weight), with 20–24% cellulose, as well as 7–8% protein and 1–2% lignin (Foster et al. 2001). According to Micard et al. (1996), SBP has a high arabinose content of 20.9% of the dry weight of the SBP, compared to 1.1% mannose and 1.7% xylose. This probably points to the presence of an arabinose-based polysaccharide such as arabinan. The presence of lignin is considered one of the most important stumbling blocks for the effective enzymatic hydrolysis of lignocellulose substrates (Merino and Cherry 2007; Varnai et al. 2010). Therefore it has become a general practice to pretreat lignocellulose substrates through some chemical pretreatment step, such as acid or alkaline hydrolysis and steam explosion (See reviews by Hendricks and Zeeman 2009; Sun and Cheng 2002). Depending on the type of pretreatment, the lignin portion may be removed or simply disrupted and in some cases the hemicellulose fraction may also be degraded during pretreatment (Hendricks and Zeeman 2009). Enzymatic degradation of cellulose has been considered the most important as most strains of *Saccharomyces cerevisiae* are only able to ferment glucose (Himmel et al. 2007). However, enzymatic degradation of hemicellulose has become more important to improve the overall yield of sugars which can also be fermented into bioethanol using other microorganisms (Dien et al. 2003; Fortman et al. 2008; Merino and Cherry 2007). As the hemicellulose fraction of SBP is very significant, a pretreatment method was therefore chosen that would disrupt the lignin without degrading the hemicellulose, namely lime pretreatment (Kumar and Wyman 2009).

Lime pretreatment using slake lime (calcium hydroxide) is often employed as it is inexpensive and can be recovered through neutralising to insoluble calcium carbonate and regeneration through lime kiln technology (Kaar and Holtzapple 2000). The optimal lime pretreatment conditions for lignin removal in SBP has not been reported in literature; thus the conditions of lime load, temperature and time were investigated in this study. Pretreatment conditions were evaluated by using three hemicellulase enzymes to enzymatically act on the various pretreated SBP fractions. The resulting activity (as total reducing sugars produced) was considered to be a measure of the pretreatment conditions that caused the greatest enhancement of hydrolysis.

Synergy between enzymes exists when the combination of enzymes displays a higher activity than the sum of the individual activities on the same substrate. It is a ratio that measures the cooperative interaction between enzymes in the degradation of a substrate and the ability of an enzyme to enhance the activity of another enzyme. A study of synergistic interactions assists in determining the areas of recalcitrance in a substrate and the mechanism by which enzymes cooperate to overcome this.

ArfA is an α-l-arabinofuranosidase, a non-cellulosomal family 51 glycosyl hydrolase from *C. cellulovorans* (Kosugi et al. 2002a). Apart from arabinofuranosidase activity, ArfA also has high activity on arabinoxylan and arabinan. XynA is an *endo*-xylanase, a family 11 glycoside hydrolase from the cellulosome of *C. cellulovorans* (Kosugi et al. 2002b). XynA also contains an acetyl xylan esterase module that contributes to deacetylation of acetylated substrates and displays a synergy with the *endo*-xylanase catalytic domain (Kosugi et al. 2002b). ManA is an *endo*-mannanase, a cellulosomal enzyme from *C. cellulovorans* and is classified as a family 5 glycosyl hydrolase with narrow substrate specificity (Tamaru and Doi 2000).

Several synergy studies have previously been completed on ArfA and XynA (Kosugi et al. 2002a; Koukiekolo et al. 2005); however, the *C.* *cellulovorans* mannanase A (ManA) has not been studied in combination with ArfA and XynA. The role of mannanases in the degradation of lignocellulose substrates has only received limited attention. Studies have indicated that an *endo*-mannanase acted synergistically in the hydrolysis of a lignocellulose substrate, namely sugarcane bagasse (Beukes et al. 2008; Beukes and Pletschke 2010, 2011). However, whether it may contribute in a similar manner to the degradation of other lignocellulose substrates has not been investigated. Studies have suggested that addition of an *endo*-xylanase in an enzyme cocktail greatly enhanced the ability of cellulases to hydrolyse a substrate, even where the xylan is only present in very low concentrations (Garcia-Aparicio et al. 2007). Therefore we wanted to investigate whether this may also be the case for a lignocellulose substrate containing low levels of mannan.

The aim of this work was therefore to optimise the lime pretreatment of SBP and then to assess the synergistic interaction (degree of synergy) of the three *C. cellulovorans* hemicellulases (ArfA, ManA and XynA), using SBP as substrate.

## Materials and methods

### Expression and purification of ArfA, ManA and XynA

Cloned genes of *arfA* (AY128945) (Kosugi et al. 2002a), *manA* (AF132735) (Tamaru and Doi 2000) and *xynA* (AY604045) (Kosugi et al. 2002b) were kindly donated by Prof. Roy Doi (University of California Davis, CA). Purification of ArfA, ManA and XynA was carried out according to the method described in Beukes et al. (2008). The efficiency of the purification was determined by electrophoresis of purified protein samples on a 12% SDS–PAGE gel (Laemmli 1970). The activity of the purified protein samples was confirmed using commercial substrates as described below. Substrates used for each enzyme were birchwood xylan for XynA, locust bean gum for mannanase activity and *p*-nitrophenyl-α-l-arabinofuranoside for ArfA.

### Protein analysis

The Bradford protocol (Bradford 1976) was utilised to determine the protein concentration using bovine serum albumin (BSA) as a suitable standard.

### Enzymatic activity: reducing sugar analysis

Hemicellulase activity was assayed in 50 mM sodium citrate buffer (pH 5.5) at 40 °C using the DNS method described by Miller (1959). Assays were performed in triplicate and the released reducing sugars (as d-xylose equivalents) were reported in units (U) of activity, with 1 U defined as the amount of hemicellulase liberating 1 μmol of reducing sugar per minute.

### Substrate preparation

The fresh sugar beet was stored at −20 °C upon arrival. The size of the pulp was reduced by chopping it into small pieces and then homogenising with a Waring blender. Multiple wash and filter steps were performed to ensure no residual reducing sugars were present on the surface of the sugar beet. The filtrate was monitored until no reaction with the DNS assay was observed. At this stage the sugar beet was described as ‘sugar beet pulp’ (SBP). Air-drying of the SBP for 24 h was performed at room temperature and the SBP was stored in an air-tight container.

### Optimisation of lime pretreatment

A 2^3^ factorial design with central replicate points, with the factors lime loading, time and temperature, was utilised to determine the optimal conditions for a slake lime pretreatment. Individual runs were carried out on 4 g of dried SBP. Wash and filter steps were completed to remove residual lime. The filtrate was tested to ensure that no residual sugars remained present on the SBP after pretreatment. The SBP was air-dried for 24 h at room temperature after completion of pretreatment.

The extent to which different pretreatment conditions were able to enhance enzymatic hydrolysis was determined using an enzyme assay containing equal concentrations (5 μg/ml) of ArfA, ManA and XynA in 50 mM sodium citrate (pH 5.5) for 24 h at 37 °C. The design matrix response was noted as the total reducing sugars (TRS), released as a result of hydrolysis using the three hemicellulases. The DNS assay was used to measure TRS with the highest levels indicating optimal pretreatment conditions. A one-way analysis of variance (ANOVA) with Design-Expert^®^ 6.0.4 software was utilised to examine the pretreatment data.

### Scanning electron microscopy (SEM)

Samples of the SBP before and after lime treatment were prepared by the Rhodes University Electron Microscopy Unit. The untreated and lime pretreated SBP samples were air dried at room temperature. Sample preparation for SEM included a sputter coating of gold (Balzers Union Sputtering Device) on the various SBP samples and subsequent examination on SEM.

### Optimisation of hemicellulase synergy

Synergy assays were set up with combinations of the three enzymes at different ratios with the total enzyme concentration always amounting to 40 μg/ml. Each enzyme was added at concentrations of 0.0, 12.5, 25.0, 37.5, 50.0, 62.5, 75.0, 87.5 and 100.0% of the total protein concentration. Triplicate reactions were conducted in 50 mM sodium citrate (pH 5.5) at 40 °C for 5 days. Enzyme activity was measured using the DNS method described above. The degree of synergy that was obtained in the synergy studies was calculated by dividing the actual activities of the recombinant enzymes obtained with the enzyme assays with the sugar beet pulp, by the theoretical sum of the enzyme activities of individual enzymes, i.e.,

## Results and discussion

### Enzyme purification

ArfA, ManA and XynA were purified to a single band on an SDS–PAGE gel and identity confirmed through activity assays on commercial substrates (data not shown). Specific activities for ArfA, ManA and XynA were calculated as 72.68, 2.4 and 11.07 U/mg, respectively.

### Lime pretreatment

The statistical model was a 2 × 2 × 2 factorial design with the factors lime loading, time and temperature. The response of the design matrix, used to evaluate optimal pretreatment conditions, was the release of total reducing sugars (TRS, μmol reducing sugar per gram of SBP over 24 h) as a result of enzyme activity from the hydrolysis of the SBP using ArfA, ManA and XynA (Table 1). An equal concentration (5 μg/ml) of each of the hemicellulases was used for hydrolysis. The highest levels of TRS was found to be 42.4, which resulted from a lime loading of 0.4 g/g SBP and a pretreatment time of 36 h at a temperature of 40 °C. With similar conditions for lime loading and pretreatment time but with the highest temperature (70 °C), TRS was much lower at 6.3, but it is not clear why the increased temperature had this effect.

**Table 1:** Total Reducing Sugars (TRS) resulting from the action of ArfA, ManA and XynA on pretreated SBP for each factorial run

Run no.	Pretreatment conditions			
	Lime (g/g SBP)	Time (h)	Temp (°C)	TRS ^a^
1	0.1	12	40	0.9
2	0.4	12	40	8.7
3	0.1	36	40	3.8
4	0.4	36	40	42.4
5	0.1	12	70	13.9
6	0.4	12	70	15.1
7	0.1	36	70	16.4
8	0.4	36	70	6.3
9	0.25	24	55	11.0
10	0.25	24	55	18.7
11	0.25	24	55	30.6

^a^Average of triplicate values for each run

TRS = μmol of reducing sugar released by enzyme action per gram of SBP per 24 h

One-way analysis of variance (ANOVA) verified the model with a significant *F*-value of 8.61 (Table 2). A confidence level of 95% was chosen; thus the terms A (lime loading), B (time of pretreatment), AC (lime loading and temperature), BC (time and temperature) and ABC (lime loading, time and temperature) were all identified as significant as the probability values fell below 0.05. Term AC, representing lime loading and temperature, had the highest *F*-value at 23.44 indicating that these two conditions displayed the highest significance. However, temperature (C) had a high *p* value of 0.7255 thus confirming the discrepancy of the temperature effect. A curvature *F*-value of 7.79 showed that there was significant curvature within the model. The triplicate central replicate points of the design matrix were included to give curvature and there is only a 1.07% chance that a curvature *F*-value could be related to noise.

**Table 2:** ANOVA for the optimisation of lime pretreatments

Source	Sum of squares	Degrees of freedom	Mean square	*F*-value	Probability > *F*
Model	2774.25	7	396.32	8.61	<0.0001
(A) Lime	498.71	1	498.71	10.83	0.0033
(B) Time	322.37	1	322.37	7.00	0.0148
(C) Temperature	5.82	1	5.82	0.13	0.7255
AB	134.78	1	134.78	2.93	0.1012
AC	1079.40	1	1079.40	23.44	<0.0001
BC	646.47	1	646.47	14.04	0.0011
ABC	627.04	1	627.04	13.62	0.0013
Curvature	358.53	1	358.53	7.79	0.0107

**Fig. 1:**
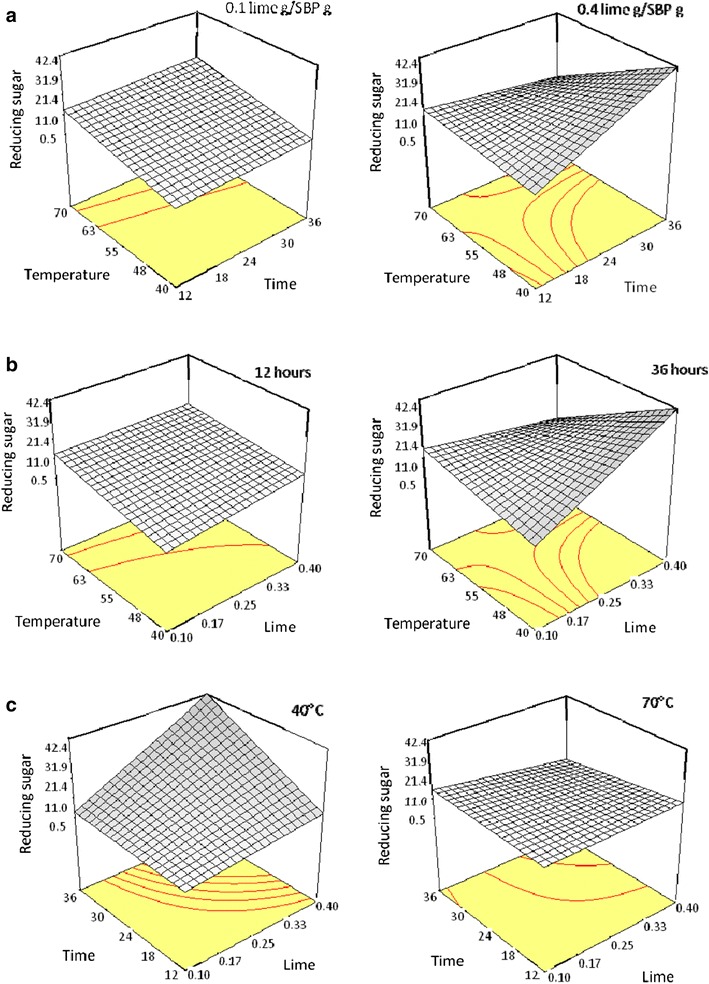
The effect of lime loading (**a**), duration (**b**) and temperature (**c**) during pretreatment of SBP on the TRS as a result of enzyme activity of a combination of ArfA, ManA and XynA (5 μg/ml each). The TRS recorded is displayed as released reducing sugar on the *z*-axis

Three-dimensional perturbation plots were used to compare the three different parameters for pretreatment on resulting enzyme activity. Figure 1a represents the effect of the concentration of lime loading on the enzyme activity, while Fig. 1b represents the effect of duration of pretreatment and Fig. 1c the effect of temperature during pretreatment on enzyme activity.

In Fig. 1a, the *x*-axis (time) and *y*-axis (temperature) were consistent with the pretreatment conditions, the *z*-axis (reducing sugars) represents the response to the enzyme assays run subsequently to the pretreatment. A significant increase in activity (as TRS) with a increase in lime loading was observed (from 0.1 g lime/g SBP to 0.4 g lime/g SBP, see Fig. 1a). The highest activity observed for 0.1 g lime/g SBP, which resulted in TRS of 16.4, was compared to 42.4 for 0.4 g lime/g SBP. Figure 1a displayed a fairly flat gradient at a low concentration of lime (0.1 g lime/g SBP) and changes in temperature or time had little effect in increasing the TRS. Temperature had a relatively greater effect than time at this low concentration of lime, as at 40 °C activity released 0.9 TRS (at 12 h) and 3.8 TRS (at 36 h) compared to 13.9 TRS (at 12 h) and 16.4 TRS (at 36 h) at 70 °C. With a lime loading of 0.4 g lime/g SBP, a three-factor interaction was observed with an increase in activity and TRS from 8.7 to 42.4 (12 to 36 h) while the temperature was kept constant at 40 °C.

The three-dimensional plots in Fig. 1b and c confirm the results from Fig. 1a. Figure 1b, investigating the effect of temperature, clearly demonstrates that the longer pretreatment time of 36 h resulted in the highest enzyme activity (42.4 TRS) compared to an enzyme activity of 15.1 TRS at 12 h. The effect of temperature (Fig. 1c) indicated that at low temperature, lime loading and time of pretreatment were the most important factors. At 70 °C, these factors did not appear to have the same impact (Fig. 1c).

The factors lime loading and incubation time produced significant responses within the design model. The third factor, temperature, was not significant with a *F*-value of 0.13 (Prob > *F* 0.7255). Industrially, a lower temperature is more favoured as there is a lower energy demand, resulting in lower costs, and with high temperatures a pressure vessel would be required (Wyman et al. 2005). Thus the effect of temperature actually posed an advantage. Therefore, the optimal conditions from this study of 40 °C, 0.4 g lime/g SBP and 36 h were used as the optimal pretreatment conditions for all subsequent experiments. As the SBP contains quite low amounts of lignin (1–2%), optimal enhancement of hydrolysis by pretreatment appeared to require a much lower severity than found with other substrates.

### Effect of lime pretreatment on structure of SBP using SEM

**Fig. 2:**
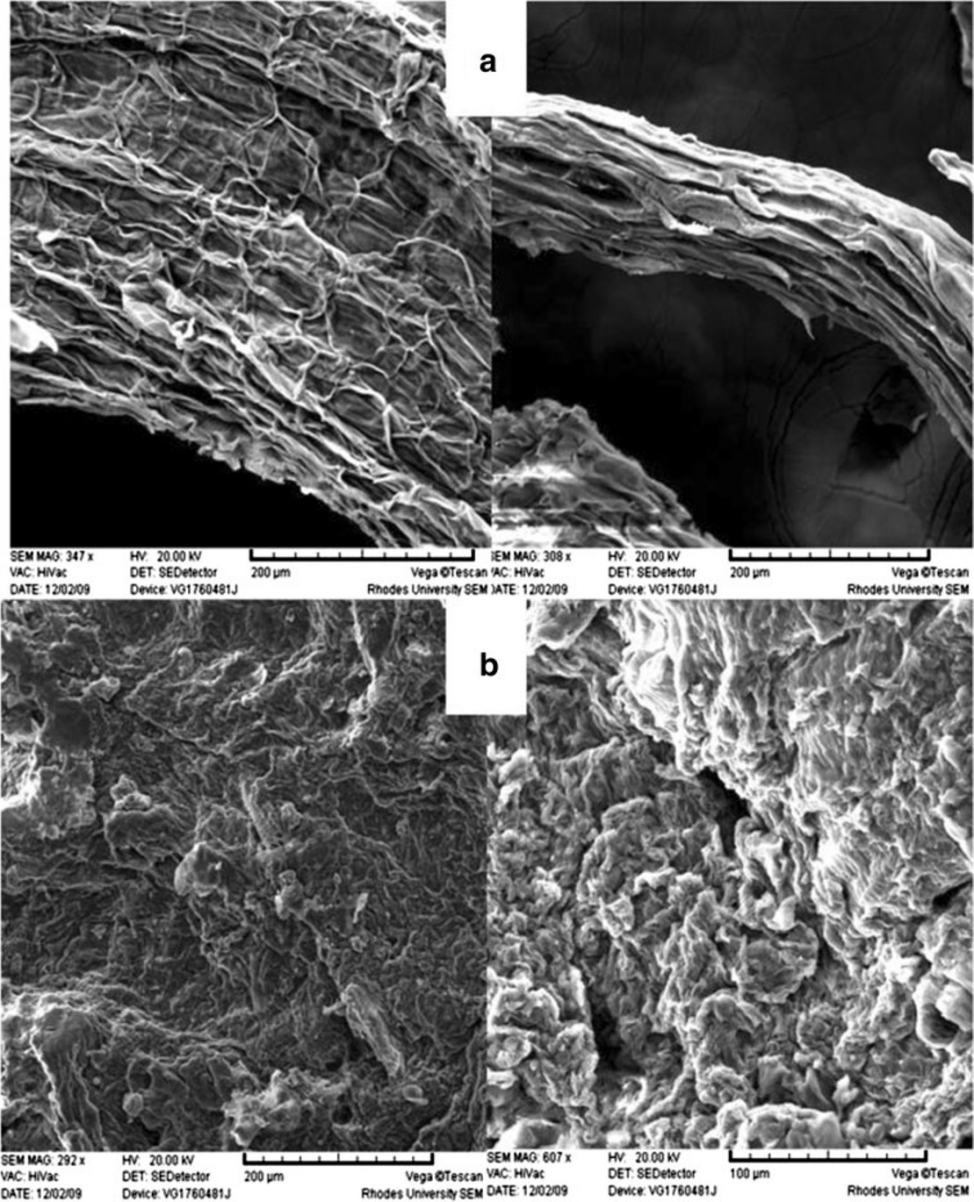
SEM micrographs of SBP before (**a**) and after pretreatment (**b**) demonstrate the disruption of fibres, visible in (**a**) to give a more amorphous structure in (**b**)

The scanning electron micrographs in Fig. 2 display the impact of the lime pretreatment on the SBP. The untreated pulp fibres (Fig. 2a) had a structured architecture and the fibres were clearly visible. However, after pretreatment, the structure of the SBP appears to become more amorphous and the fibres were indistinguishable (Fig. 2b), indicating that fibre disruption had taken place.

### Synergy studies with *C. cellulovorans* ArfA, ManA and XynA on pretreated SBP

**Fig. 3:**
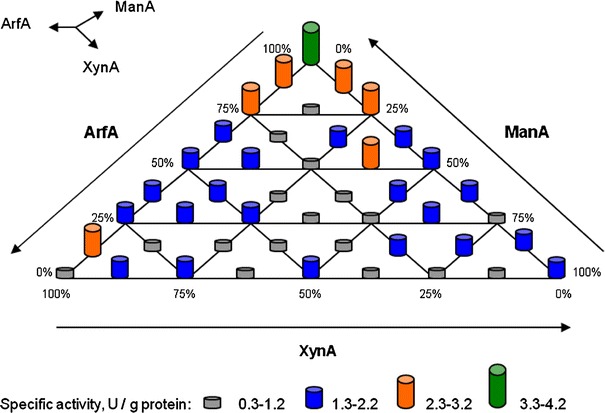
Specific activities of ArfA, ManA and XynA with SBP. The relative specific activities are indicated using the code provided. The three enzymes were mixed in various ratios of protein concentration to a total of 40 μg/ml. Each enzyme is displayed on the axes as a percentage of total protein and the *arrows* indicate the directions of axes for each enzyme concentration

**Fig. 4:**
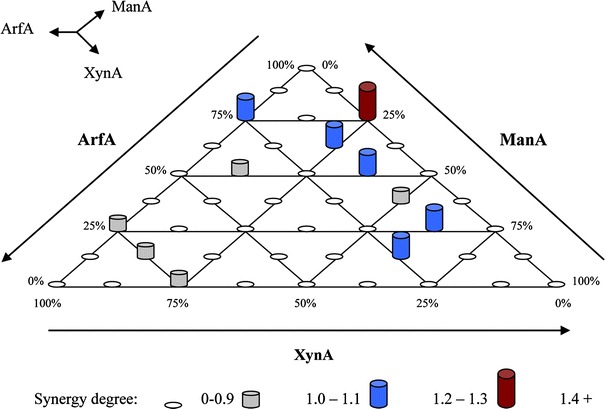
Degrees of synergy of ArfA, ManA and XynA with SBP. The relative degrees of synergy are indicated using the code provided. The three enzymes were mixed in various ratios as a percentage of the total protein of 40 μg/ml in every assay. Each enzyme is displayed on the axes as a molar percentage and the *arrows* indicate the directions of axes for each enzyme concentration. The degree of synergy that was obtained in the synergy studies was calculated by dividing the sum of the actual activities of the recombinant enzymes obtained with the enzyme assays with the sugar beet pulp, by the theoretical sum of the recombinant enzyme activities

Various combinations of the three hemicellulases were tested on the optimised lime pretreated SBP. The results in Fig. 3 are depicted in a three-dimensional triangle that can be read according to the tri-directional arrowed key. Relative concentrations of the enzymes are depicted on the three axes, with the combinations which produced the highest activity indicated. Figure 4 depicts the same three enzyme combinations, but reflects the degree of synergy displayed by the different ratios of enzymes in the assay, rather than the actual activity.

The highest activity was noted at 100% ArfA (4.2 U/g protein), indicating that the arabinofuranosidase contributed extensively to hydrolysis of this substrate. As ArfA also displays arabinase activity, it was able to act on the high concentration of arabinan in the SBP and convert it to increased levels of reducing sugars. The surrounding combinations had a slightly lower activity ranging between 2.3 and 3.2 U/g protein, indicating that the higher activity levels favoured higher ArfA concentrations. Achievement of high hydrolysis yields would also require the addition of cellulases within an optimised enzyme mixture, but the results clearly indicate that an arabinase/arabinofuranosidase would be the most important hemicellulase to use in combination with cellulases.

Synergistic associations, which revealed cooperative interaction between the three hemicellulases, were shown to be optimal (i.e. exhibit the highest degree of synergy) at 75% ArfA: 25% ManA (Fig. 4). The low mannan composition of the SBP explains the relatively low requirement for ManA activity. But the fact that synergy between these two enzymes was established, indicated that the presence of ManA enhanced the activity of the ArfA, possibly due to the mannan in the substrate hampering access of ArfA to the arabinan in the SBP. The presence of XynA did not increase activity at a synergistic level, despite a similar content of XynA to ManA in SBP. This may suggest that the xylan was not closely associated with mannan or arabinan in the SBP.

The results obtained for specific activity and degrees of synergy using the various combinations of hemicellulases therefore highlighted the high requirement for ArfA, although ManA enhanced activity of ArfA. The low mannan content in SBP as reported by Micard et al. (1996) did not suggest that an *endo*-mannanase would be required for the synergistic degradation of SBP. However, it is possible that the role of mannanases have thus far been understated in the degradation of complex substrates.

A similar observation was made in the study by Beukes et al*.* (2008) on the synergistic degradation of sugarcane bagasse by enzymes from *C. cellulovorans*, where it was observed that mannanase was required for high synergy on this substrate even though bagasse only contained low levels of mannan. It was therefore suggested by Beukes et al. (2008) that the mannan fibres were obstructing XynA and EngE from acting on the xylan and cellulose fibres.

Some indication of the importance of arabinases/arabinofuranosidases in SBP hydrolysis has been suggested in literature. Spagnuolo et al*.* (1999) reported that incubation of beet pulp with two arabinases (α-l-arabinofuranosidase and *endo*-arabinase), used singularly or in combination, led to the hydrolysis of arabinan, which produced arabinose in the hydrolysate. In addition, Van der Veen et al. (1991) found that sugar beet pulp was an inducer of arabinase activity in *Aspergillus niger* N400. Three arabinan degrading enzymes (α-l-arabinofuranosidase A, α-l-arabinofuranosidase B and *endo*-arabinase) were produced. Utilisation of SBP as a substrate for bioethanol production should therefore include arabinase/arabinofuranosidase, as well as mannanase, in an enzyme consortium for enzymatic degradation.

This study provides information on the structure of SBP and the manner in which the hemicellulose fibres are associated within the pulp. While ArfA proved to be the most important hemicellulase for degradation of the hemicellulose fraction of SBP, the unexpected result was the role of ManA in this process. Based on the very low mannan content in SBP, 1.1% according to Micard et al. (1996), mannanase would have appeared insignificant for the degradation of SBP hemicellulose. However, it was demonstrated that ManA had a clear synergistic relationship with the ArfA and therefore should be included in an enzyme consortium for degradation of SBP. SBP has the potential to be utilised as a second generation biofuel source and optimised lime treatment and exposure to arabinase/arabinofuranosidase activity hold the key to the efficient enzymatic hydrolysis of SBP.
